# 8p23.2-pter Microdeletions: Seven New Cases Narrowing the Candidate Region and Review of the Literature

**DOI:** 10.3390/genes12050652

**Published:** 2021-04-27

**Authors:** Ilaria Catusi, Maria Garzo, Anna Paola Capra, Silvana Briuglia, Chiara Baldo, Maria Paola Canevini, Rachele Cantone, Flaviana Elia, Francesca Forzano, Ornella Galesi, Enrico Grosso, Michela Malacarne, Angela Peron, Corrado Romano, Monica Saccani, Lidia Larizza, Maria Paola Recalcati

**Affiliations:** 1 Istituto Auxologico Italiano, IRCCS, Laboratory of Medical Cytogenetics and Molecular Genetics, 20145 Milan, Italy; m.garzo@auxologico.it (M.G.); l.larizza@auxologico.it (L.L.); p.recalcati@auxologico.it (M.P.R.); 2 Department of Biomedical, Dental, Morphological and Functional Imaging Sciences, University of Messina, 98100 Messina, Italy; annapaola.capra@unime.it (A.P.C.); silvana.briuglia@unime.it (S.B.); 3 UOC Laboratorio di Genetica Umana, IRCCS Istituto Giannina Gaslini, 16147 Genova, Italy; chiarabaldo@gaslini.org (C.B.); michelamalacarne@gaslini.org (M.M.); 4 Child Neuropsychiatry Unit—Epilepsy Center, Department of Health Sciences, ASST Santi Paolo e Carlo, San Paolo Hospital, Università Degli Studi di Milano, 20142 Milan, Italy; mariapaola.canevini@unimi.it (M.P.C.); angela.peron@unimi.it (A.P.); monica.saccani@asst-santipaolocarlo.it (M.S.); 5 Medical Genetics Unit, Città della Salute e della Scienza University Hospital, 10126 Turin, Italy; rachele.cantone@unito.it (R.C.); egrosso@cittadellasalute.to.it (E.G.); 6 Unit of Psychology, Oasi Research Institute-IRCCS, 94018 Troina, Italy; felia@oasi.en.it; 7 Clinical Genetics Department, Guy’s & St Thomas’ NHS Foundation Trust, London SE1 9RT, UK; Francesca.Forzano@gstt.nhs.uk; 8 Laboratory of Medical Genetics, Oasi Research Institute-IRCCS, 94018 Troina, Italy; ogalesi@oasi.en.it; 9 Human Pathology and Medical Genetics, ASST Santi Paolo e Carlo, San Paolo Hospital, 20142 Milan, Italy; 10 Division of Medical Genetics, Department of Pediatrics, University of Utah School of Medicine, Salt Lake City, UT 84132, USA; 11 Unit of Pediatrics and Medical Genetics, Oasi Research Institute-IRCCS, 94018 Troina, Italy; cromano@oasi.en.it

**Keywords:** 8p23.2-pter microdeletion, 8p23.3, chromosomal microarray analysis (CMA), critical microdeletion region (CR), candidate region, small deletions, *ARGHEF10*, *DLGAP2*, developmental delay, behavior disorder

## Abstract

To date only five patients with 8p23.2-pter microdeletions manifesting a mild-to-moderate cognitive impairment and/or developmental delay, dysmorphisms and neurobehavioral issues were reported. The smallest microdeletion described by Wu in 2010 suggested a critical region (CR) of 2.1 Mb including several genes, out of which *FBXO25*, *DLGAP2*, *CLN8*, *ARHGEF10* and *MYOM2* are the main candidates. Here we present seven additional patients with 8p23.2-pter microdeletions, ranging from 71.79 kb to 4.55 Mb. The review of five previously reported and nine Decipher patients confirmed the association of the CR with a variable clinical phenotype characterized by intellectual disability/developmental delay, including language and speech delay and/or motor impairment, behavioral anomalies, autism spectrum disorder, dysmorphisms, microcephaly, fingers/toes anomalies and epilepsy. Genotype analysis allowed to narrow down the 8p23.3 candidate region which includes only *DLGAP2*, *CLN8* and *ARHGEF10* genes, accounting for the main signs of the broad clinical phenotype associated to 8p23.2-pter microdeletions. This region is more restricted compared to the previously proposed CR. Overall, our data favor the hypothesis that *DLGAP2* is the actual strongest candidate for neurodevelopmental/behavioral phenotypes. Additional patients will be necessary to validate the pathogenic role of *DLGAP2* and better define how the two contiguous genes, *ARHGEF10* and *CLN8*, might contribute to the clinical phenotype.

## 1. Introduction

Deletions of the distal region of the short arm of chromosome 8 have been frequently reported in the literature as either isolated terminal/interstitial deletions or terminal deletions associated with more proximal duplications [1]. These rearrangements are mainly due to the architecture of the 8p23.1 region, characterized by the presence of two olfactory receptor gene clusters and defensin repeats, named REPD (repeat-distal) and REPP (repeat-proximal), that mediate chromosomal rearrangements through a U-type exchange mechanism [2,3,4,5,6].

Conversely, large deletions including the 8p23.1 region and isolated microdeletions of the more distal 8p23.2-pter region have rarely been reported in patients displaying various clinical features, including developmental delay (DD)/intellectual disability (ID), microcephaly, mildly dysmorphic features, language delay and/or behavioral problems [7]. Microdeletions of this region are hardly ever recorded in the database of genomic variants (DGV) which collects copy number variants (CNVs) in healthy control samples [8]. 

To date only five patients carrying 8p23.2-pter microdeletions have been characterized by chromosomal microarray (CMA) analysis in the literature and other cases have been reported in the DECIPHER database [7,9,10,11]. Furthermore, the smallest microdeletion described by Wu et al. in 2010 suggested a possible critical region (CR) of 2.1 Mb in 8p23.3 [7,11]. The CR comprises several genes out of which the OMIM genes *FBXO25*, *DLGAP2*, *CLN8*, *ARHGEF10* and *MYOM2*, most of which share a role in neural differentiation and function [12,13,14,15,16,17], are main candidates for DD/ID, microcephaly and neurobehavioral disorders.

Here we report seven new patients with interstitial microdeletions included in the 8p23.2-pter region which partially overlap with the previously mentioned CR [7,11]. Phenotypic evaluation of our patients, compared to that of five previously reported patients and additional nine obtained from the DECIPHER database, confirmed a shared clinical phenotype, mainly characterized by ID/DD, including language and speech delay and/or motor impairment, behavioral anomalies/attention deficit and hyperactive disorder (ADHD), autism spectrum disorder (ASD) and dysmorphisms. The analysis of microdeletion extensions enabled us to point out a candidate region, more restricted compared to the previously proposed CR, and to propose a genotype-to-phenotype correlation for a few core clinical signs. Moreover, possible mechanisms accounting for the clinical variability across microdeletion carriers are herein discussed. 

## 2. Materials and Methods

The seven patients described underwent standard and molecular cytogenetics analyses as routine diagnostic procedures in five different Italian Cytogenetics Laboratories. Informed consent signed by the patients’ parents, adult patients themselves and healthy adult carriers was provided. Chromosome analysis was performed on peripheral blood lymphocytes using QFQ banding techniques on metaphase chromosomes obtained by standard procedures. Array based comparative genomic hybridization (array-CGH) analyses were performed using: Bluegnome 4 × 180 K CytoChip Oligo ISCA, resolution ~25 kb (Patient 1); Agilent SurePrint G3 Human CGH+SNP Microarray kit 4 × 180 K, resolution ~25 Kb (Patients 2 and 7); Agilent SurePrint G3 Oligo ISCA v2.0 4 × 180 K, resolution ~25 kb (Patient 4); Agilent SurePrint G3 Human CGH Microarray kit 8 × 60 K, resolution ~100–150 kb (Patients 3, 5 and 6). Nucleotide designations were assigned according to the hg19/GRCh37 assembly of the human genome. BAC-FISH (Bacterial artificial chromosome – Fluorescence in situ hybridization) experiments with the RP11-666I19 BAC clone were performed according to Lichter et al. 1988 [18] with minor modifications. FISH analyses with chromosome 8 subtelomeric-specific probes (ToTelVysion, Vysis, Downers Grove, IL, USA) were performed according to manufacturer’s instructions. All coding exons of the *CLN8* gene were analyzed by direct sequencing of polymerase chain reaction (PCR) products using standard protocols. Patients 1, 2 and 4 underwent Leiter-3, ADOS (Autism Diagnostic Observation Schedule), VABS II (Vineland Adaptive Behavior Scales-II), ABC-2 (Movement Assessment Battery for Children 2), PEP-3 (PsychoEducational Profile 3) and ADI-R tests. Patient 5 underwent WISC-III (Wechsler Intelligence Scale for Children-III), Leiter-R and ADOS tests; Patient 7 underwent WISC-IV, Leiter-3 and CPM (Coloured Progressive Matrices) tests. In patients 3 and 6, ID and DD assessments have been performed through clinical evaluation by experienced neurologists.

Protocols provided by the companies have been followed with no modifications. 

Consent for publication of research studies were obtained from all individuals.

## 3. Results

### Cytogenomic and Phenotypic Characterization of Seven Novel 8p23-Pter Microdeletion Patients: Cross-Comparison to Previously Reported Patients

**Figure 1:**
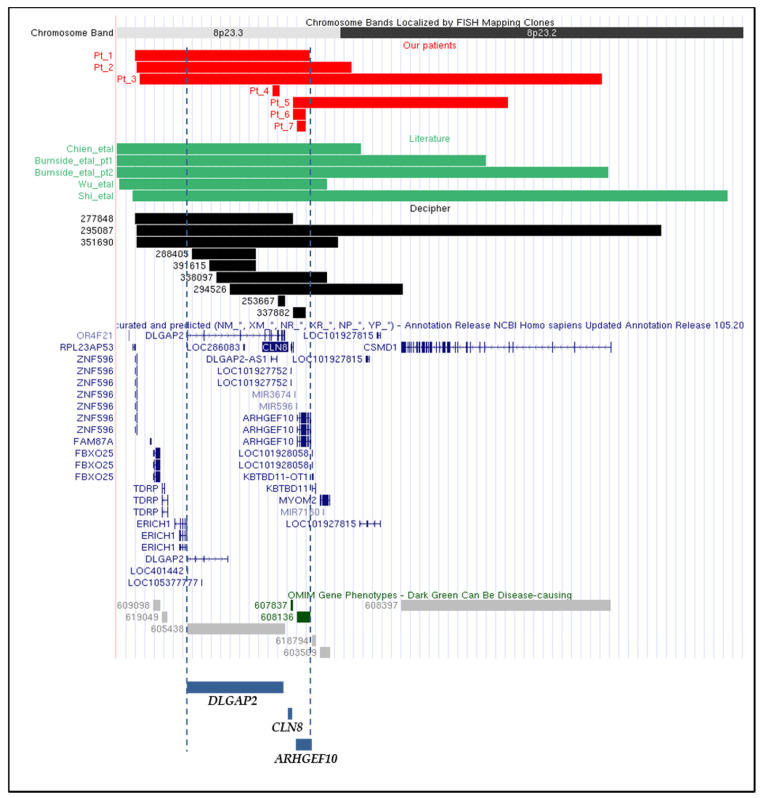
Physical map of the 8p23.2-pter region (nucleotides 1 to 6,000,000 GRCh37/hg19) adapted from the UCSC Genome Browser [20]: differently colored bars indicate the different genomic regions involved in the microdeletions of: our seven patients (RED bars), patients reported in the literature (GREEN bars), patients described in the DECIPHER database (BLACK bars). All curated and predicted NCBI RefSeq genes included in this region are annotated. Genes included in the candidate region are indicated below with BLUE bars. DARK GREEN bars point to OMIM-disease genes, whereas LIGHT GREY bars indicate genes not associated to any OMIM phenotype [17].

Our study includes seven patients, six males and one female, with age at diagnosis ranging from 2 to 38 years. CMA was performed for all seven patients and the results are provided in Figure 1 and Table 1. Of note, Patients 3, 5 and 6 have been extrapolated from a cohort of 5110 Italian patients, collected by the Cytogenetics/Cytogenomics working group of the Italian Society of Human Genetics (SIGU) and processed by CMA as reported [19]. Patients 1, 2, 4 and 7 were collected by the same SIGU working group afterwards. Patient 7 corresponds to DECIPHER patient 383055. Additional approaches used were: standard cytogenetics analysis, that for Patient 3 revealed a mosaic karyotype 45,XY,-8[8]/46,XY,r(8)(p23.2q24.3) [42], and targeted FISH analysis using the 8p subtelomeric probe and BAC probe RP11-666I19, to confirm the microdeletion in Patient 1 (data not shown). Array-CGH analysis on Patient 3 genomic DNA didn’t reveal either imbalances involving 8q or mosaic monosomy of chromosome 8. However, the declared detection rate of the array-CGH platform used does not guarantee to identify mosaicism under the rate of 30%. Furthermore, given that the 123 kb deletion of Patient 6, which involves only the *CLN8* and *ARHGEF10* genes, did not manifest any effect in the carrier brother, direct sequencing analysis of polymerase chain reaction (PCR) products was performed on the patient’s genomic DNA in order to check if eventual unmasking of a recessive *CLN8* mutation on the non-deleted allele could be responsible for the patient’s phenotype. Sequence changes in *CLN8* exons were ruled out (data not shown). The inheritance pattern was precluded for Patient 1, since he was adopted, but could be determined in five of the six remaining patients: the microdeletion arose *de novo* in Patients 3 and 4, was transmitted to Patient 2 by his apparently healthy mother and to Patient 5 by his father, which presented a milder phenotype. Moreover, we presume that the microdeletion was inherited also by Patient 6, because his unaffected brother carried the same microdeletion (Table 1). The extent of the microdeletions ranged from the 71.79 kb deletion detected in Patient 4, which contained only the *DLGAP2* gene, to the 4.55 Mb deletion of Patient 3, comprising 9 coding genes (Figure 1 and Table 1). 

Table 2 lists the genes comprised in the patients’ microdeletions, their OMIM entry, when available, the encoded proteins and their assessed, putative or unknown main role, their association to disease, if applicable, and all inherent references. The last column of the table provides the pLI (probability of Loss of function Intolerance) score, a metric which ranges between 0 and 1 and measures the extent of tolerance of a given gene to heterozygous loss-of function variants [21,22], a parameter suitable to explore the role of genes contributing to the phenotype of microdeletion syndromes.

All genes map to 8p23.3, except the most centromeric *CSMD1*, which maps to 8p23.2. Noncoding genes localized at 8p23.2-pter are not included in the list because knowledge of their function is limited and most of them are not annotated in OMIM. 

Out of the 11 protein coding genes encompassing the deletion intervals of our patients, *DLGAP2* and *ARHGEF10* appear to be, according to their expression profile and the assessed role in neural morphogenesis, differentiation and function [13,14,15,16], *bona fide* candidates for neurodevelopmental disorders. Interestingly, *DLGAP2,* a “synaptic” gene associated with autism [13], is the only gene lost in Patient 4, who carries the smallest microdeletion (71.79 kb) and presents behavioral issues (Table 1); *ARHGEF10* is the only gene lost in the second smallest microdeletion (83.33 kb), found in Patient 7, who also displays a hyperkinetic behavior (Table 1). *ARHGEF10,* together with *CLN8*, also falls within the 123 kb deletion of patient 6 who has motor coordination problems and epilepsy (Table 1). Both *DLGAP2* and *ARHGEF10* also map within the deletion intervals of the remaining four patients. The *CLN8* gene is involved in lipid synthesis, transport, or sensing [23]. The *FBXO25* and *KBTBD11* genes have cell cycle regulation and metabolic functions [24,25], while *TDRP* is involved in spermatogenesis [26] and *MYOM2* encodes a strictly muscular-related protein [27]. Even more limited is the knowledge on the remaining *OR4F21*, *ZNF596*, *ERICH1* and *CSMD1* microdeletion genes. 

RNA-seq data from the GTEx Portal show a high level of expression in the whole brain for *DLGAP2*, *KBTBD11* and *CSMD1*, and in some nervous system tissues for *ZNF596* (cerebellum) and *CLN8* (spinal cord) [28].

Comparison of the microdeletions of our seven patients with those of the five patients reported in the literature highlighted a similar extent and an identical gene content between the microdeletion of our Patient 2 and the literature smallest microdeletion which allowed to identify the CR [7,11]. It also allowed to point out the presence in Patient 5 of another microdeletion, similar in length, but staggered. In addition, while the largest microdeletion in our series (Patient 3) stands between those reported in Patients 1 and 2 by Burnside [9], the increasingly smaller deletion intervals of 1.72 Mb, 123 kb, 83.33 kb and 71.79 kb in our Patients 1, 6, 7 and 4, respectively, provided the tool to address a genotype-phenotype analysis by cross evaluating the clinical signs displayed by our patients and the reported microdeleted patients. As detailed in Table 1, growth delay combined to mild cognitive impairment and/or ASD manifestations and a few dysmorphic signs, particularly microcephaly, emerged as core features of both the seven newly characterized patients and the five previously reported ones. However not all the listed signs were present across patients and their combination was variable, apparently not dependent on the deletion size (Table 1). In order to better define the variable clinical expressivity of 8p23.2-pter microdeletion, 9 additional individuals from the DECIPHER database [29] were included in the genomic and clinical comparison, summing up to a total of 21 individuals (Table 3). DECIPHER patients 396027, 396034, 396025, 396345 and 396353, referred to the same laboratory and carrying 8p23.3 microdeletions with the same extension and identical end points, were excluded from our analysis as we could not discriminate between their possible familial relationship and a longitudinal study of the same individual at different ages. DECIPHER patient 394930 has been excluded because he carries the same 8p23.3 microdeletion and a large pathogenic rearrangement involving chromosome 12. 

Evaluation of the expanded cohort of 21 8p23.2-pter microdeleted patients confirmed that the main phenotype is variably compounded by ID (13/21), DD (9/27), including language and/or speech delay (7/21) and motor impairment (5/21), behavioral anomalies/ADHD (8/21), ASD (6/21), dysmorphisms (8/21), microcephaly (6/21), fingers/toes anomalies (6/21), and epilepsy (4/21) (Table 3).

## 4. Discussion

We overviewed seven new patients carrying microdeletions sized from 71.79 kb to 4.55 Mb encompassing the 8p23.2-pter region. All microdeletions partially overlap with the CR including the *FBXO25*, *DLGAP2, CLN8, ARHGEF10* and *MYOM2* genes, that have been previously pointed out by Wu et al. and Shi et al. [7,11]. Loss of these genes may be of pathogenic relevance as 8p23.2-pter CNVs are rarely reported in the healthy population according to the DGV database [8]. Patient 3 should be considered more prudently compared to other patients since conventional cytogenetic analysis revealed a low-level mosaicism for chromosome 8 monosomy, non excludable by array-CGH analysis, making difficult to assess its contribution to the clinical phenotype.

Our patients have been referred for variable clinical features consistent with those described in 5 previously reported microdeleted patients (Table 1) and in 9 patients recorded in the DECIPHER Database, summing up to a total of 21 individuals (Table 3). The small number of the evaluated patients so far and the lack of records for specific signs in a few cases does not allow to calculate an accurate frequency for each considered feature. In addition, since age at diagnosis spans between <1 to 38 years, incompletely evaluated late onset features in some patients must be also taken into account as contributors to the phenotypic variability. However, considering the entire composite cohort, ID and DD, including speech and/or motor delay, are present in the majority of individuals. Other common features in decreasing trend are behavioral anomalies/ADHD, ASD, dysmorphisms and microcephaly. We also observed fingers/toes anomalies in few individuals, whereas epilepsy/seizure was reported only occasionally. 

Overall, microdeletions arose *de novo* in 5 patients and were inherited in 6 out of the 21 patients herein surveyed. The familial cases, including one paternal transmission (our Patient 5), three maternal transmissions (our Patient 2, and DECIPHER patients 288405 and 391615) and two unknown transmissions (brother of Patient 6 and DECIPHER patient 277848) attest the relatively mild overall phenotype of even large microdeletion carriers and their spared reproductive fitness. This observation values the provision of genetic counseling and the careful collection of familial history also in those that are apparently sporadic cases. Recognition of the unaffected brother of Patient 6 and the apparently asymptomatic mother of Patient 2 highlights a possible defect of penetrance which may lead to underestimate 8p23.2-pter microdeletion carriers. However, inaccurate clinical evaluation of these asymptomatic carriers cannot be excluded since some patients are characterized by very mild clinical features. Additional cases are required to strengthen the incomplete penetrance of 8p23.2-pter microdeletions.

Although sharing deleted genes, the 21 different microdeletions analyzed do not appear to share common breakpoints, a finding which may account for non recurrent microdeletions at the population level [30,31]. The different rearrangement sizes, genomic extents, and breakpoint positions suggest non-homologous end joining (NHEJ) and replication mechanisms as the main causative drivers of the described microdeletions [32]. The analysis of the deletion intervals does not highlight a single overlapping region. However, the smallest microdeletions identified in Patients 4, 6 and 7, as well as in DECIPHER patients 253667, 288405, 337882 and 391615, point out two CRs, one including *DLGAP2* and the other *ARHGEF10* (Figure 1, Table 1 and Table 3).

*ARHGEF10* (Rho guanine nucleotide exchange factor 10) encodes a guanine-nucleotide exchange factor (GEF) for the Rho family of GTPase proteins (RhoGEFs) [33]. The function of *ARHGEF10* has not been completely elucidated, but recent evidence suggests it could be specifically linked to the membrane trafficking pathway [14,15,34]. A neuronal role for *ARHGEF10* has emerged with the identification of a familial Thr332Ile heterozygous mutation, which causes constitutive activation of GEF, and which is associated to autosomal dominant non-progressive slow nerve conduction velocities and thin peripheral nerve myelinization without clinical phenotype (OMIM #608236) (Table 2) [34,35]. *ARHGEF10* variants have been also associated to schizophrenia and to Charcot-Marie Tooth disease type 1A (CMT1A) [36,37,38,39]. In accordance with this presumptive role, DECIPHER patient 368323, carrying an SNV (single nucleotide variant) of uncertain clinical significance in *ARHGEF10*, mainly showed global developmental delay, postnatal microcephaly, encephalopathy and seizure, that are typical features of 8p23.2-pter patients (Table 1 and Table 3). In addition, *ARHGEF10* is included among autism genes with a suggestive evidence score in the SFARI database (score 3) [40].

*DLGAP2* (discs, large Drosophila homolog-associated protein 2) belongs to the DLGAP family of scaffolding proteins acting in the post-synaptic density (PSD), a highly specialized matrix involved in transmission of neuronal signals across the synaptic junction [16,41]. Several studies pointed out an association between *DLGAP2* and ASD, obsessive-compulsive disorder (OCD) and schizophrenia [10,11,13,42,43,44]. In particular, two duplications of *DLGAP2* have been recently reported in an ASD cohort [45]. Accordingly, *DLGAP2* is also present in the SFARI Gene database with a suggestive evidence score of 3 [40]. Most importantly, and conversely to *ARHGEF10*, *DLGAP2* is a brain-specific expressed and dosage-sensitive gene with a pLI score of 1 (Table 2) [21], indicating it may contribute to the clinical phenotype of 8p23.2-pter microdeleted patients. 

**Figure 2:**
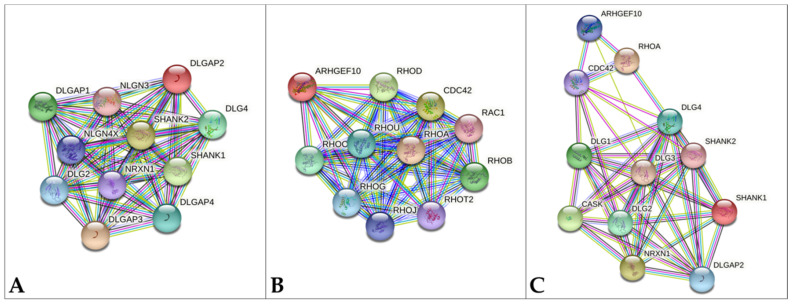
Protein-protein interaction in STRING database [57]. (**A**) *DLGAP2* network, (**B**) *ARHGEF10* network (**C**) *ARHGEF10* and *DLGAP2* common network. The top ten interactors for each gene are shown. Colored lines indicate different kinds of interactions. Blue: known interaction from curated databases; violet: known interaction experimentally determined; green: predicted interaction for gene neighborhood; red: predicted interaction for gene fusion; dark blue: predicted interaction for gene co-occurrence; yellow: interaction based on text mining; black: interaction based on gene-expression; light blue: interaction based on protein homology.

In 2017 Shi et al. proposed *DLGAP2* as candidate gene for microcephaly [7], given that polymorphisms in *DLGAP2* have been associated to orbital frontal cortex (OFC) white matter volume alterations in OCD patients [46]. Moreover, DLGAP2 interacts with NRXN1, a pre-synaptic cell-adhesion molecule (Figure 2A) and CASK, another scaffolding protein (Figure 2C) and mutations in both *NRXN1* and *CASK* are associated to neurodevelopmental and neurocognitive disabilities and microcephaly [47,48,49]. Disruption of the CASK-Neurexin interaction was recently proposed to be strictly linked to microcephaly onset in *CASK*-mutated patients [50]. Weakening of the DLGAP2-NRXN1 interaction upon *DLGAP2* haploinsufficiency and interfering with the NRXN1-CASK interaction might concur to microcephaly onset also in 8p23.2-pter patients. In our cohort, the presence of microcephaly only in Patient 3, which carries a 4.55 Mb microdeletion encompassing 9 genes and a mosaic karyotype with a chromosome 8 monosomy cell line, does not allow to confirm *DLGAP2* as the main candidate gene for microcephaly. In addition, microdeleted patients in the literature displaying microcephaly were shown to carry large deletions, leaving open the implication of other 8p23.2-pter genes. In particular, *ARHGEF10* is involved in neuronal morphogenesis and DECIPHER Patient 368323, which carries an *ARHGEF10* SNV, presents microcephaly; *CSMD1* and *KBTBD11* are brain-specific expressed genes, and *CSMD1* is also dosage-sensitive. The strict connection between brain genes and microcephaly is well-documented in the literature [51,52,53]. 

Both *DLGAP2* and *ARHGEF10* could be potential candidates for behavioral disorders, in accordance with the phenotype of *Dlgap2*^-/-^ and *Arhgef10*^-/-^ mice [54,55,56]. Accordingly, our Patients 2, 4, 5 and 7, which carry 8p23.2-pter microdeletions involving *DLGAP2* and/or *ARHGEF10*, are characterized by hyperactivity/hyperkinetic behavior and/or aggressiveness (Table 1).

Based on its dosage-sensitivity, the brain-specific expression and the *de novo* origin of Patient’s 4 microdeletion, *DLGAP2* seems to be a stronger candidate than *ARHGEF10* for both the neurodevelopmental and behavioral phenotypes. The causative role of *ARHGEF10* appears weaker since none of the microdeletions involving *ARHGEF10* without *DLGAP2* (Patients 5, 6 and 7), were demonstrated to be *de novo*. Moreover, the asymptomatic brother of Patient 6 may be interpreted as a sign of reduced penetrance, as above mentioned, but could also be evidence against causality.

**Figure 3:**
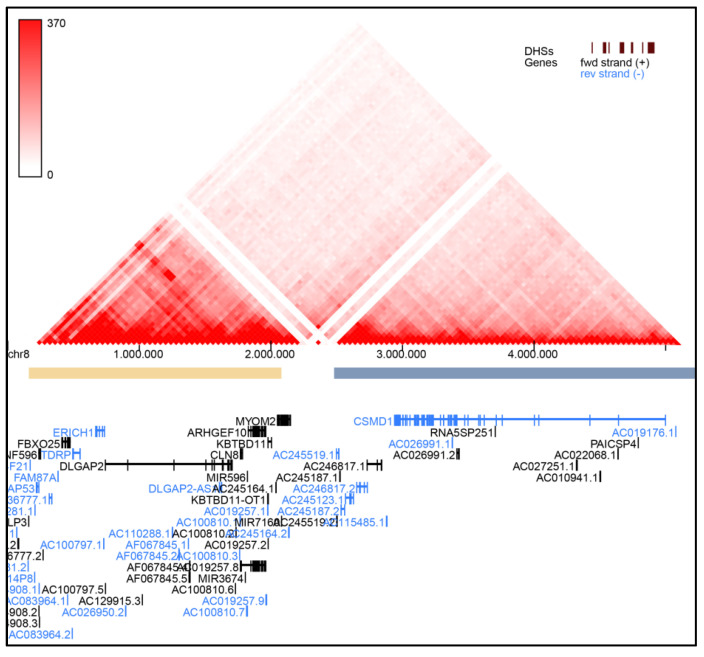
Topological associated domains (TADs) in 8p23.2-pter region based on Hi-C data performed by YUE Lab website [72].

Therefore, for these latter patients, pathogenic mechanisms other than *ARHGEF10* loss, are possible. In particular, the microdeletion could impact the expression of genes flanking the deleted region, such as the nearby *DLGAP2*, through a position effect mechanism or by disruption of the topological associated domains (TADs) architecture. Indeed, in the 8p23.2-pter region two adjacent TADs are present with a boundary localized between *MYOM2* and *CSMD1* (Figure 3). For example, the microdeletion of Patient 5, which involves the boundary but not *DLGAP2*, could cause neurodevelopmental and behavioral consequences due to *DLGAP2* deregulation. Assessment of *DLGAP2* expression levels in patients which deletion breakpoints map to the boundary of the two main 8p23.2-pter architectural domains might furnish preliminary insights.

Despite all the above-mentioned considerations, *ARHGEF10* deserves to be evaluated in further microdeleted patients because of its documented role in neuronal morphogenesis and its joined action with *DLGAP2* on post-synaptic scaffolding proteins. The family of post-synaptic scaffolding proteins includes members of the SHANK, DLG and DLGAP subfamilies, which interact in post-synapses [16]. As shown in Figure 2A, DLGAP2 interacts with SHANK1–2, DLG2-4 and DLGAP1-2-3. Disruption of the network of scaffolding proteins alters the homeostasis of the PSD, contributing to various neuropsychiatric conditions, such as autism spectrum disorders, intellectual disability, behavioral problems, schizophrenia, and bipolar disorders [16,58,59,60,61,62]. 

At the same time, *ARHGEF10* defects, which cause alterations in Rho GTPases function, could have an indirect effect on the activity of scaffolding proteins (Figure 2C). The STRING database shows the interaction between ARHGEF10 and small GTPases, such as RhoA and CDC42 (Figure 2B), which play an important role in regulating the shape of most neuron cell types and interact with scaffolding proteins, such as SHANK [63,64]. A role for GTPases in neurodevelopmental disorders, especially autism [59,62] is attested by the association of *RhoA* and *CDC42* genes to ASD [65,66,67]. 

Based on the clinical phenotype and on the CR [7,11], we pointed out for the first time, motor impairment as another clinical feature associated with 8p23.2-pter microdeletions and linked it to *CLN8* and *ARHGEF10,* the only genes included in the 123 kb deletion of our Patient 6, which showed fine and gross coordination problems. The *CLN8* (Ceroid-lipofuscinosis neuronal 8) gene belongs to the TLC (TRAM, Lag1, and CLN8 homology domains) superfamily and has a role in the biosynthesis and metabolism of lipids and in the protection of proteins from proteolysis [23]. Homozygous genetic alterations of *CLN8* are associated with neuronal ceroid lipofuscinosis (NCL) (OMIM #600143), a genetically heterogeneous group of neurodegenerative disorders mainly characterized by seizures and progressive neurological deterioration, which result in dementia, ataxia, visual failure, and various forms of abnormal movement. Despite the fact that a mutation of *CLN8* in the non deleted allele of Patient 6 has been ruled out and that, to date, a clinical phenotype has never been reported for *CLN8* heterozygous deletion/mutation, a higher risk for motor impairment should be assessed in future *CLN8* heterozygous deleted patients.

Since also *CLN8*, which maps between *DLGAP2* and *ARHEGF10*, seems to be potentially clinically relevant, the two CRs could be merged into a single candidate region, including *DLGAP2*, *CLN8* and *ARHGEF10*, that could be a valuable landmark for future investigations of additional 8p23.2-pter microdeleted patients. This region is more restricted compared to the previously proposed CR [7,11].

As regards the pronounced clinical variability observed in 8p23.3-pter patients it may result from their genetic heterogeneity, linked to microdeletions size and gene content. Beyond those involved in the actually restricted candidate region, other genes less frequently included in 8p23.2-pter microdeletions may cooperate to the clinical spectrum. Examples are *CSMD1* in 8p23.2, a dosage-sensitive gene associated to ASD, schizophrenia and epilepsy [68,69,70] and *FBXO25,* recently associated to neuropsychiatric disorders [71]. Both genes are not included in most of the microdeletions reported to date, but they might act as phenotypic modifiers in patients with microdeletions spanning until the 8p23.2 or 8p23.3 regions, respectively. Although we focused our attention on coding genes, the 8p23.2-pter region includes several noncoding genes for different types of regulatory RNAs, whose microdeletion could also have an effect on the clinical phenotype. The contribution to the clinical phenotype of 8p23.3-pter patients of several other coding or noncoding genes, remains to be identified. Dysmorphisms for example were reported only in patients carrying larger microdeletions, not involving only *DLGAP2* and/or *ARHGEF10,* and this coincidence needs to be confirmed. 

In addition, several genomic and epigenetic mechanisms may contribute to the phenotypic variability of 8p23.2-pter patients. 

First, the genetic make up may influence the severity of the phenotype. The presence of other CNVs or mutations in the genome could have an additive effect on the clinical phenotype, as exemplified by Patients 2 and 7 additional CNVs, namely 5p15.2 microdeletion and 6q26 microduplication. Although these CNVs could be classified as likely benign according to international criteria, there could be a pathogenic effect in an 8p23.2-pter deleted background.

Second, epigenetic mechanisms may also play a role. In particular, *DLGAP2* is predicted to be a maternally imprinted gene [73]. Although it seems to be expressed from both alleles in whole brain, the effect of its deletion on the severity of the clinical phenotype could be dependent on its parental origin. In patients carrying the microdeletion on the paternal allele, the effect of *DLGAP2* loss could be worst. In favor of this hypothesis, the *DLGAP2* microdeletions identified in the OCD and the ASD cohorts are both paternally inherited [42,45]. In order to support this hypothesis, the parental origin of the deleted allele should be checked, even in patients with a *de novo* origin of the microdeletion. 

## 5. Conclusions

In conclusion, this study expands the limited number of described patients carrying microdeletions involving the 8p23.2-pter region. Comparison of the 7 novel patients with the 5 previously reported and the additional 9 recorded in DECIPHER confirms the association to a variable clinical phenotype, mainly characterized by ID/DD, including language and/or speech delay and motor impairment, behavioral anomalies/ADHD, ASD, dysmorphisms and microcephaly. The analysis of small microdeletions allowed us to dissect the previously proposed CR, delineating a new candidate region including three 8p23.3 genes: *DLGAP2, CLN8* and *ARHGEF10*. Out of these three genes, *DLGAP2* is the more robust candidate due to its pLI score and its brain-specific expression, which account for the neurodevelopmental-specific effect of its loss. Investigation of novel patients will permit to validate the pathogenic role of *DLGAP2*, define how the two contiguous genes *CLN8* and *ARHGEF10* might contribute to the clinical phenotype and determine the role of other 8p23.2-pter genes.

## Figures and Tables

**Table 1:** Clinical and molecular genetics data of 8p microdeleted patients described in the present work and in the literature.

	**Present Work Patients**							**Literature Patients**					
	**Patient 1**	**Patient 2**	**Patient 3**	**Patient 4**	**Patient 5**	**Patient 6**	**Patient 7**	**Shi et al., 2017** [7]	**Burnside et al., 2013 pt1** [9]	**Burnside et al., 2013 pt2** [9]	**Chien et al., 2010** [10]	**Wu et al., 2010** [11]	Total Number of Patients
Age at diagnosis	13	4	16	2	13	38	12	5	2	4	12	1	
Sex	M	M	M	M	M	M	F	M	F	M	M	F	
Coordinates (GRCh37/hg19)	chr8:176475-1892812	chr8:191530-2308985	chr8:221611-4767606	chr8:1531691-1603483	chr8:1731454-3846288	chr8:1731454-1853939	chr8:1774139-1857463	chr8:158048-6004205	chr8:1- 3623904	chr8:1-4832134	chr8:1-2400000	chr8:21000-2067000	
Size	1.72 Mb	2.12 Mb	4.55 Mb	71.79 kb	2.1 Mb	123 kb	83.33 kb	6.0 Mb	3.6 Mb	4.8 Mb	2.4 Mb	2.1 Mb	
Inheritance		Maternal	*de novo*	*de novo*	paternal (father with learning disability and stuttering)	inherited (present in the unaffected brother)		*de novo*	*de novo*	*de novo*			
Coding gene(s) content	*ORF4F21*, *ZNF596*, *FBXO25*, *TDRP*, *ERICH1*, ***DLGAP2***, ***CLN8***, ***ARHGEF10***	*ORF4F21*, *ZNF596*, *FBXO25*, *TDRP*, *ERICH1*, ***DLGAP2***, ***CLN8***, ***ARHGEF10***, *KBTBD11*, *MYOM2*	*FBXO25*, *TDRP*, *ERICH1*, ***DLGAP2***, ***CLN8***, ***ARHGEF10***, *KBTBD11*, *MYOM2*, *CSMD1*	***DLGAP2***	***CLN8, ARHGEF10,*** *KBTBD11, MYOM2, CSMD1*	***CLN8, ARHGEF10***	***ARHGEF10***	*ORF4F21*, *ZNF596*, *FBXO25*, *TDRP*, *ERICH1*, ***DLGAP2***, ***CLN8***, ***ARHGEF10***, *KBTBD11*, *MYOM2*, *CSMD1*	*ORF4F21*, *ZNF596*, *FBXO25*, *TDRP*, *ERICH1*, ***DLGAP2***, ***CLN8***, ***ARHGEF10***, *KBTBD11*, *MYOM2*, *CSMD1*	*ORF4F21*, *ZNF596*, *FBXO25*, *TDRP*, *ERICH1*, ***DLGAP2***, ***CLN8***, ***ARHGEF10***, *KBTBD11*, *MYOM2*, *CSMD1*	*ORF4F21*, *ZNF596*, *FBXO25*, *TDRP*, *ERICH1*, ***DLGAP2***, ***CLN8***, ***ARHGEF10***, *KBTBD11*, *MYOM2*	*ORF4F21*, *ZNF596*, *FBXO25*, *TDRP*, *ERICH1*, ***DLGAP2***, ***CLN8***, ***ARHGEF10***, *KBTBD11*, *MYOM2*	
ID	mild ID		mild ID		mild DD at 3y. IQ = 91 at 13y			+			+	+	6/12
DD		+	-	-	+	+	+	+	+	+	+	+	9/12
Language and speech delay	+				+		+	+	+	-	+		6/12
Motor impairment	Motor instability, dyspraxia	Motor instability, balance/coordination problems, limb hypotonia			−	Fine and gross coordination problems		Balanced/coordination problems	Balanced/coordination problems				5/12
ASD		+		+	+		−	+	−	−	+		5/12
Behavioral abnormalities/ ADHD		Hyperkinetic behavior, irritability		Hyperkinetic behavior, ADHD	Hyperactivity, aggressiveness, impulsiveness, stereotype behavior		Hyperkinetic behavior, hyperactivity, attention deficit, aggressiveness	ADHD	−	−	ADHD		6/12
Microcephaly			+				−	+	Microcephaly, brachycephaly	+	−	+	5/12
Fingers/ toes anomalies			Shortened 4th toe of left foot, bilateral broad 1st toe, finger hyperlaxity		Bilateral clinodactyly of the 5th finger					Nail hypoplasia			3/12
Dysmophisms	+ (not specified)	−	Bitemporal narrowing, hypotelorism, prograthism, premature graying of hair	−	Low-set ears, narrow palpebral fissures, thin vermillion of the upper lip	−		low-set ears with bilateral prominence of the antitragus, epicanthal folds and a long philtrum	Mild maxillary, flattening medial epicanthal folds with upslanting palpebral fissures, preauricular pit and bilateral prominence of the antitragus	Palpable metopic ridge, upslanting palpebral, fissures, nystagmus, aniridia, low-set and posteriorly rotated ears with overfolded helices, small upturned nose, and downturned corners of the mouth	−	Hypertelorism, long philtrum, malformed ears	7/12
Epilepsy			−		−	+	+				+		3/12
Other	Myopia, strabismus, skeletal anomalies	Xerosis cutis, skin anomalies, mild hepatomegaly, cerebral parenchymal anomalies	Short stature, eutrophic skin,	Hippocampal anomalies, heart anomalies	Perinatal distress, jaundice, flat feet, frequent respiratory infections	Scoliosis	Emmetropia, scoliosis, horshoe kidney, hypereosinophily, teeth cavities, hypotyroidism			Coartaction of the aorta			
Other CNVs (GRCh37/ hg19)/ additional genetic data		5p15.2 microdeletion coordinates 12260192-12849583 (maternal)	45,XY,-8[8]/ 46,XY,r(8)(p23.2q24.3)[42]			Absence of mutations in *CLN8* coding sequence on the other allele	6q26 deletion coordinates 163274414-163399757						

The information for each patient includes: age at diagnosis, sex, 8p microdeletion coordinates (Assembly GRCh37/hg19), microdeletion size, inheritance, gene content, clinical features and other additional genetic/genomic data. The number of patients showing a clinical feature compared to the total number of patients (present work and literature patients) is indicated in the last column. Main candidate genes are displayed in bold characters. “+” means present; “−” means absent; empty cells: data not available. ADHD: attention deficit and hyperactive disorder; ASD: autism spectrum disorders; chr: chromosome; CNV: copy number variant; DD: developmental delay; ID: intellectual disability; IQ: intelligence quotient; y: years.

**Table 2:** Coding genes included in 8p23.2-pter region.

Human Gene Symbol/OMIM Entry	Gene Full Name	Main Biological Activity	Reference	OMIM Disease Association	pLI Score
*OR4F21*	Olfactory receptor family 4 subfamily F member 21	Olfactory receptor	np	np	np
*ZNF596*	Zinc finger protein 596	Transcriptional regulation	np	np	0
*FBXO25* */* *609098	F-box protein 25	Substrate-recognition component of the SCF (SKP1-CUL1-F- box protein)-type E3 ubiquitin ligase complex.	[24]	np	0
*TDRP*	Testis development related protein	Contributes to normal sperm motility	[26]	np	0
*ERICH1*	Glutamate-rich 1	Unknown	np	np	0
*DLGAP2* /*605438	Discs, large (Drosophila) homolog-associated protein 2	Role in the molecular organization of synapses and neuronal cell signaling. Adapter protein linking ion channel to the sub-synaptic cytoskeleton	[16]	np	1
*CLN8* /*607837	Ceroid-lipofuscinosis, neuronal 8 (epilepsy, progressive with mental retardation)	Lipid synthesis, transport, or sensing	[23]	Ceroid lipofuscinosis 8, AR (# 600143) Ceroid lipofuscinosis, neuronal, 8, Northern epilepsy variant, AR (# 610003)	0
*ARHGEF10* */* *608136	Rho guanine nucleotide exchange factor (GEF) 10	Membrane trafficking and neural morphogenesis	[14,15]	?Slowed nerve conduction velocity, AD (# 608236)	0
*KBTBD11* /*618794	Kelch repeat and BTB (POZ) domain containing 11	Protein degradation, thereby affecting differentiation, homeostasis, metabolism, cell signaling, and oxidative stress response	[25]	np	0.06
*MYOM2* /*603509	Myomesin 2	Major component of the vertebrate myofibrillar M band	[27]	np	0
*CSMD1* /*608397	CUB and Sushi multiple domains 1	Role in central nervous system development	[12]	np	1

Gene symbol, OMIM entry, full name, main known biological activity and relative reference, OMIM disease association if applicable and pLI (probability of a gene being intolerant to variation causing loss of gene function) score from GnomAD are reported. “np” stands for: not present [17,21].

**Table 3:** Clinical and molecular genetics data of 8p microdeleted patients described in the DECIPHER database [29].

	**DECIPHER Patients**									TOTAL NUMBER OF PATIENTS
	**253667**	**277848**	**288405**	**294526**	**295087**	**338097**	**337882**	**351690**	**391615**	
Age at diagnosis	8	5	nd	nd	10	nd	<1	13	nd	
Sex	F	M	nd	M	F	M	M	M	M	
Coordinates (GRCh37/hg19)	chr8:1588355-1656051	chr8:176814-1734772	chr8:738801-1369613	chr8:1111157-2908829	chr8:191560-5353580	chr8:974820-2067679	chr8:1731454-1853939	chr8:194617-2170951	chr8:907664-1368668	
Size	68 kb	1.56 Mb	633 kb	1.80 Mb	5.16 Mb	1.09 Mb	122 kb	1.98 Mb	463 kb	
Inheritance		inherited (parent with the same phenotype)	maternal			*de novo*			maternal	
Coding gene(s) content	***DLGAP2***	*ZNF596*, *FBXO25*, *TDRP*, *ERICH1*, ***DLGAP2, CLN8***	***DLGAP2***	***DLGAP2***, ***CLN8***, ***ARHGEF10***, *KBTBD11, MYOM2, CSMD1*	*ZNF596*, *FBXO25*, *TDRP*, *ERICH1*, ***DLGAP2***, ***CLN8***, ***ARHGEF10***, *KBTBD11, MYOM2, CSMD1*	***DLGAP2***, ***CLN8***, ***ARHGEF10***, *KBTBD11, MYOM2*	***CLN8***, ***ARHGEF10***	*ZNF596*, *FBXO25*, *TDRP*, *ERICH1*, ***DLGAP2***, ***CLN8***, ***ARHGEF10***, *KBTBD11, MYOM2*	***DLGAP2***	
ID	+	Learning disability	+	+	+	+		+		13/21
DD										9/21
Language and speech delay									+	7/21
Motor impairment										5/21
ASD			+							6/21
Behavioral problems/ADHD						+			ADHD	8/21
Microcephaly	+									6/21
Fingers/toes anomalies	Clinodactyly of the 5th finger			Short phalanx of fingers, sandal gap			Short middle phalanx finger			6/21
Dysmophisms	Epicanthus									8/21
Epilepsy			+							4/21
Other	Spotty hyperpigmentation						Coarctation of the aorta			
Other CNVs (GRCh37/hg19)/ additional genetic data							4q26 duplication coordinates 119606253-120003254			

The information for each patient includes: age at diagnosis, sex, 8p microdeletion coordinates (assembly GRCh37/hg19) and size, inheritance, gene content, clinical features and other additional genetic/genomic data. The number of patients showing a clinical feature compared to the total number of patients (present work, literature and DECIPHER patients) is indicated in the last column. Main candidate genes are displayed in bold characters. “+” means present; “−” means absent; empty cells: data not available. ADHD: attention deficit and hyperactive disorder; ASD: autism spectrum disorders; chr: chromosome; CNV: copy number variant; DD: developmental delay; ID: intellectual disability.

## Data Availability

Not applicable.
